# Anæmia as a True Disease

**Published:** 1920-09-25

**Authors:** 


### ANÆMIA AS A TRUE DISEASE.

The quality of the blood and its hæmoglobin content vary widely under the influence of many different agencies, and most of these factors, apart from trauma, can, as far as the human body is concerned, be regarded as toxins. The science of medicine, which often is content with giving a name to any morbid condition, has divided anæmic conditions into primary and secondary anæmias.

### Secondary Anæmias.

Secondary anæmias are those for which a gross pathological cause can easily be found, and the term primary anæmia is usually reserved for those conditions for which the cause is obscure, and in some cases impossible to find. The secondary anæmias can almost all be regarded as toxic in origin, with the few exceptions which can be called traumatic. As examples of traumatic secondary anæmias we can mention, as the commonest types, the anæmia following extensive hæmorrhage and the anæmia following severe injuries or surgical operations, and the recovery from these types of anæmia is usually quick and uninterrupted, as the blood and the blood-forming organs have only one task to perform—*i.e.*, to provide a fresh supply of corpuscles. The other types of secondary anæmia are more difficult to overcome, as the cause is usually a toxin, and the blood-forming organs are working under the continual handicap of its effects. Some of these toxins, if detected, can be easily removed, and, with their removal, the anæmic condition soon departs, examples of such toxins being lead, syphilis, any acute septic disturbance, and unhygienic conditions of living. There are, however, other causes of secondary anæmia not so easily eradicated, and therefore the anæmia is more difficult to cure.

As such causes of a chronic secondary anæmia we might mention malignant neoplasm and tubercle. Both of these conditions creep upon their victim so insidiously that their presence is often firmly established before they are even suspected or recognised. The secondary anæmia in these cases is marked, and increases as the exciting cause becomes more firmly established. The blood prepares for a long fight, as can be seen by the relative increase of lymphocytes at the expense of the polymorphonuclear leucocytes. In these cases, as the primary cause is often impossible to eradicate, the treatment of this type of anæmia is only the treatment of symptoms as they arise.

Also, there are two anæmic conditions of which the cause is still obscure, and they are termed primary anæmias.

### Primary Anæmias.

In this respect the term primary anæmia is bad, as it seems to imply that these two conditions arise *de novo*, and it is difficult to imagine a morbid condition arising without any exciting cause at all. The two so-called primary anæmias are pernicious anæmia and chlorosis, both well-marked conditions and accompanied by a high degree of anæmia. Chlorosis is an anæmia found among young, costive girls; the anæmia is profound, but the diminution in the number of the red corpuscles is not so great. A comforting fact is that this type of anæmia reacts remarkably well to treatment, as iron, in alimentary form, acts like a specific drug in these cases. Pernicious anæmia is the most serious of all the anæmias; it has been well named a malignant disease of the blood. The underlying cause is probably some smouldering focus of septic infection acting over a long time. A careful histological examination of the blood is the best means of diagnosing this condition, and such a diagnosis should not be lightly made until all laboratory aids have been employed. The diagnosis of pernicious anæmia is a heavy sentence to pass upon any patient, as it means a long illness, and often a rapidly fatal one. At present treatment consists of eradicating sepsis and treating symptoms as they arise.

Other fairly common anæmic conditions are those in which the hæmoglobin is diminished and the leucocytes increased, and of these—leukæmias—the commonest are lymphatic leukæmia and splenomedullary leukæmia. In the former the small lymphocytes are markedly in excess, and the course is nearly always rapidly fatal. In the latter type of leukæmia the myelocytes are the predominating leucocytes, and the course of the disease is also fatal. Another leukæmia is Hodgkin's disease, which can only be satisfactorily diagnosed by an histological examination of one of the enlarged lymph glands. The conditions are all serious morbid states, as they are all fatal before very long, and all are usually accompanied with enlargement of the lymphatic glands and spleen.

Hence we can regard anæmia as being secondary, primary, or leukæmic, and the prognosis is usually good with the secondary types where the exciting cause can be removed, and bad in the cases of primary and leukæmic anæmias, as in all these cases the efficiency of the patient depends on his amount of hæmoglobin available for vital purposes.

